# let-7b and miR-126 Are Down-Regulated in Tumor Tissue and Correlate with Microvessel Density and Survival Outcomes in Non–Small–Cell Lung Cancer

**DOI:** 10.1371/journal.pone.0045577

**Published:** 2012-09-24

**Authors:** Edin Jusufović, Matija Rijavec, Dragan Keser, Peter Korošec, Eva Sodja, Ermina Iljazović, Zorica Radojević, Mitja Košnik

**Affiliations:** 1 Polyclinic for Pulmonary Diseases, Medical Center Tuzla, Tuzla, Bosnia and Herzegovina; 2 Medical Faculty, University of Tuzla, Tuzla, Bosnia and Herzegovina; 3 University Clinic Golnik, Golnik, Slovenia; 4 General Hospital Užice, Užice, Serbia; University Magna Graecia, Italy

## Abstract

Angiogenesis is a critical event in the development, progression, and spread of various human cancers, including lung cancer. Molecular mechanisms that underlie the complex regulation of angiogenic processes are poorly understood. However, an increasing body of evidence indicates miRNAs as important regulators of tumor angiogenesis. Forceps biopsies were collected from tumor tissue, surrounding tissue, and non-tumor tissue from 50 NSCLC patients. Lung tissue samples from individuals with no clinical evidence of a cancerous disease served as controls. Immunohistochemical staining for Factor VIII was used to evaluate microvessel density (MVD). TaqMan® primer-probe sets were used in quantitative real-time RT-PCR reactions to determine expression levels of let-7b, miR-126, miR-9, and miR-19a. We demonstrated significantly higher MVD and decreased expression levels of let-7b and miR-126 in tumor tissue and surrounding tissue in comparison to corresponding non-tumor tissue or lung tissue from the control group. In addition, no differences in MVD and expression levels of both miRNAs between tumor tissue and surrounding tissue from NSCLC patients were observed. Low expression of both miRNAs correlated with high MVD and worse progression-free survival and overall survival. These observations strongly suggest similar molecular alternations within tumor tissue and surrounding tissue that comprise a specific microenvironment. Low expression of let-7b and miR-126 seems to have a possible anti-angiogenic role in lung tumor tissue and significantly correlates with worse survival outcomes for lung cancer patients. Moreover, the regulation of let-7b and miR-126 expression could have therapeutic potential because it could reduce tumor angiogenesis and therefore suppress tumor growth in lung cancer patients.

## Introduction

Lung cancer, the leading cause of cancer-related deaths worldwide, is frequently diagnosed in its advanced stage; therefore chemotherapy combined with thoracic radiotherapy represents the mainstay of treatment. However, a therapy that interferes with specific molecular targets holds great promise in improving clinical outcomes in lung cancer treatment [1], [2].

The development, progression, and metastasis of various human cancers are strongly dependent on angiogenesis, a physiological process involving the generation of new blood vessels from pre-existing vasculature. A developing solid tumor is generally in an oxygen-starved or hypoxic state, which triggers the differential expression of mediators that either promote or suppress angiogenesis [3]. Angiogenic factors (e.g., the family of vascular endothelial growth factors [VEGFs] and their receptors) controlling this complex process have been the focus of efforts to identify novel therapeutic targets and to develop treatment strategies in order to inhibit angiogenesis and suppress the uncontrolled proliferation of developing tumors [2]. Therefore, elucidating the molecular mechanisms that regulate angiogenesis could be of great importance in reducing cancer-related mortality [3], [4].

MicroRNAs (miRNAs) are short (averaging 19 to 25 nucleotides long) regulatory RNA molecules with a primary function in the silencing of gene expression, which is mainly based on posttranscriptional suppression of the target mRNA by repressing translation and/or by promoting mRNA decay [5], [6]. Mature miRNAs control the expression of numerous genes that regulate fundamental biological processes including angiogenesis [6]. Many miRNAs regulate angiogenesis, although their specific roles in angiogenic process are poorly characterized. Their potential tumor-suppressive or oncogenic role in this process has been proposed in various human cancers [7]–[11]. Moreover, accumulating evidence proposes miRNAs as attractive targets for developing treatment strategies that would achieve better clinical outcomes in cancer patients [12]–[14].

Several authors demonstrated reduced expression of the let-7 miRNA family, including let-7b, in either tumor tissue or tumor cell lines when compared to their normal counterparts [13], [15], [16] suggesting that members of the let-7 family are crucial regulators of cellular processes involved in initiation and progression of various human cancers. In non-malignant cells, let-7b suppresses translation of several proteins involved in lung cancer development, some of them with a potential role in angiogenesis (e.g., C-MYC and K-RAS) [17], [18].

Another miRNA frequently down-regulated in several human cancers is miR-126 [9], [12], [19], which targets the expression of VEGFA and other molecular targets with a potential role in blood-vessel formation (e.g., VCAM1, EGFL7, and PIK3R2) [19]–[21]. Liu et al. [12] found decreased expression of miR-126 and increased expression of VEGFA in various lung cancer cell lines, and demonstrated that introduction of miR-126 into tumor cells using a lentiviral vector could down-regulate the expression of VEGFA.

Members of the miR-17-92 cluster are known to act as oncogenes in the development of lung cancer [14], [22]. It has been shown that expression of the miR-17-92 cluster is directly activated by oncogenes C-MYC and N-MYC [23], [24]. The consequential high expression of its member, miR-19a, was found to correlate with decreased expression of several anti-angiogenic factors such as thrombospondin-1 (TSP-1) and connective tissue growth factor (CTGF) [23], [25].

The information about the role of miR-9 in lung cancer development and angiogenesis is still lacking. Using microarray analysis, down-regulation of miR-9 in lung tumor tissue in comparison to corresponding non-tumor tissue was reported [15]. On the other hand, expression of miR-9 seems to be up-regulated in breast cancer, where it reduces E-cadherin expression leading to increased expression of VEGFA and promotion of angiogenesis [10].

The aim of this study was to examine the degree of vascularization determined by microvessel density as well as expression of selected miRNAs (specifically, let-7b, miR-126, miR-9, and miR-19a) in lung tumor tissue, surrounding tissue, and corresponding non-tumor tissue in patients with squamous cell lung cancer (SCC) and lung adenocarcinoma (ADC) as well as in lung tissue from control individuals without clinical evidence of a malignant disease. The relationships between miRNA expression levels and survival outcomes (PFS and OS) after 1 year follow-up were also assessed. The results of this study could improve the molecular staging of lung cancer and may offer valuable insights into identifying potential anti-angiogenic targeted therapy in lung cancer.

## Results

### Microvessel density (MVD)

**Figure 1:**
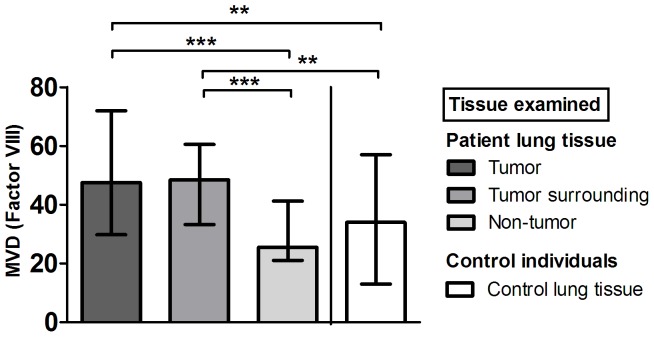
Microvessel density (MVD) assessed by Factor VIII staining. Data are presented as median with interquartile range.

**Figure 2:**
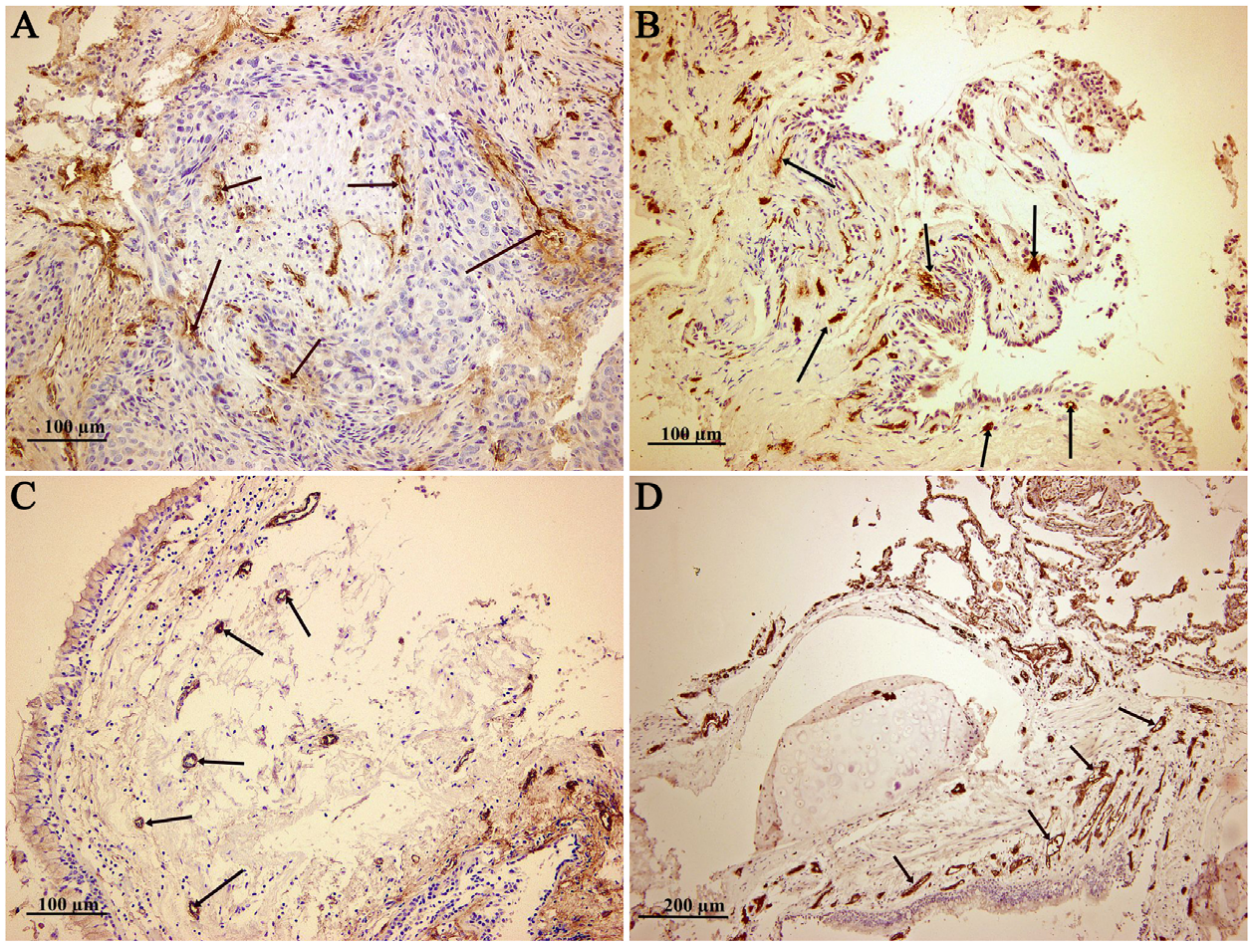
Representative cases of immunohistochemical detection of Factor VIII and microvessel density (MVD) in FFPE samples: tumor tissue (A), surrounding tissue (B), and non-tumor tissue (C) from a patient with squamous cell lung cancer, as well as lung tissue from a control individual with no pathological finding (D). Arrows indicate blood vessels.

MVD in tumor tissue (median [range]: 48 [17–123]) and surrounding lung tissue (median [range]: 49 [14–120]) was significantly higher in comparison to corresponding non-tumor tissue (median [range]: 26 [12–89]) or control lung tissue (median [range]: 34 [5–87]; Figure 1). No differences in MVD were observed between tumor tissue and surrounding tissue or when patients were stratified according to stage of disease, type of lung cancer or sex. Similarly, no differences in MVD were detected within the control group of individuals with various pathological findings (data not shown). Figure 2 presents representative case of MVD (Factor VIII) immunostaining in FFPE tissue samples.

### miRNA expression and correlations with microvessel density

Expression of selected miRNAs was determined in all tissue samples and normalized to a sample from control group. No significant correlations were observed between the expression of selected miRNAs and either clinical stage of disease, type of lung cancer or patients' sex.

**Figure 3:**
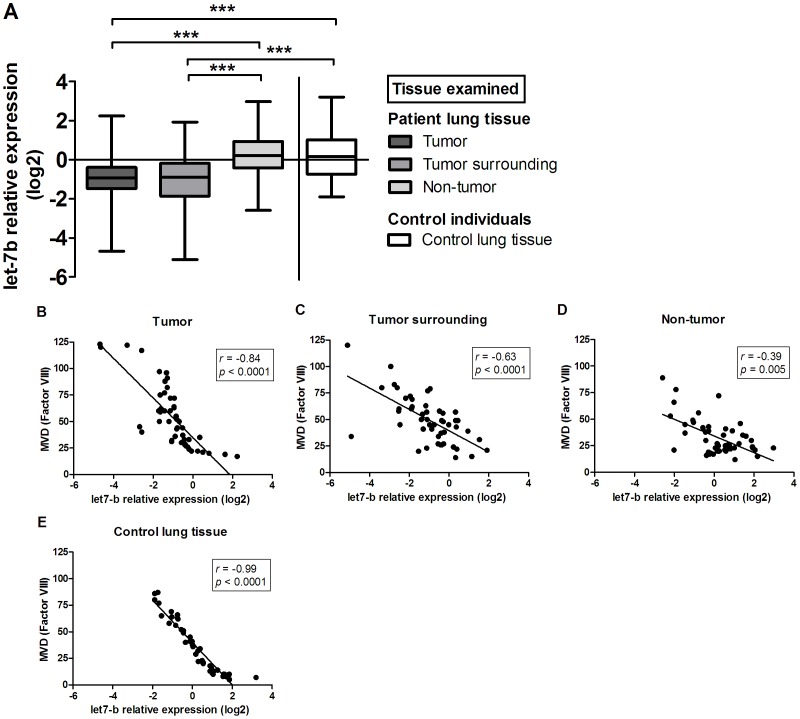
Comparative analysis of let-7b expression in lung cancer patients' tumor tissue, surrounding tissue, and non-tumor lung tissue, as well as in lung tissue from control individuals (A). Data are presented as median with range and interquartile range. Correlation between let-7b relative expression and MVD in lung cancer patients' tumor tissue (B), surrounding tissue (C), and non-tumor lung tissue (D), as well as in lung tissue from control individuals (E).

**Figure 4:**
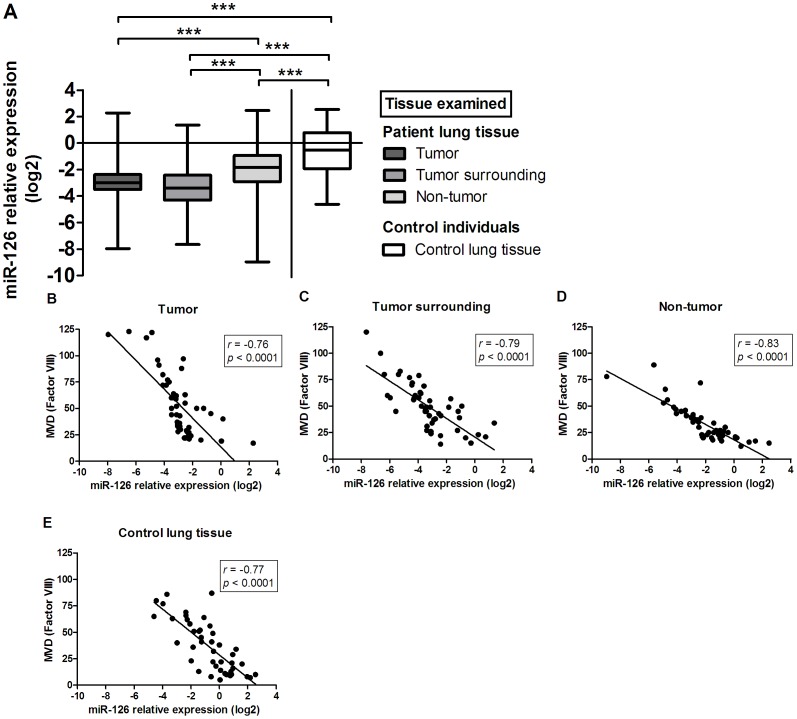
Comparative analysis of miR-126 expression in lung cancer patients' tumor tissue, surrounding tissue, and non-tumor lung tissue, as well as in lung tissue from control individuals (A). Data are presented as median with range and interquartile range. Correlation between miR-126 relative expression and MVD in lung cancer patients' tumor tissue (B), surrounding tissue (C), and non-tumor lung tissue (D), as well as in lung tissue from control individuals (E).

We found significantly reduced expression of either let-7b or miR-126 in tumor tissue and surrounding tissue when compared to corresponding non-tumor lung tissue or lung tissue from the control group (let-7b/miR-126: *p*<0.001; Figures 3A and 4A, respectively). No differences in expression of either let-7b or miR-126 were observed between tumor tissue and surrounding tissue. No differences in let-7b expression were also noted between non-tumor tissue and control lung tissue. On the other hand, significant differences in miR-126 expression were found between both tissue types. Furthermore, we found a high negative association between let-7b (Figure 3B–E) or miR-126 expression (Figure 4B–E) and MVD in all tissue samples where the overall r_S_ correlation coefficient was −0.784 and −0.752, respectively.

**Figure 5:**
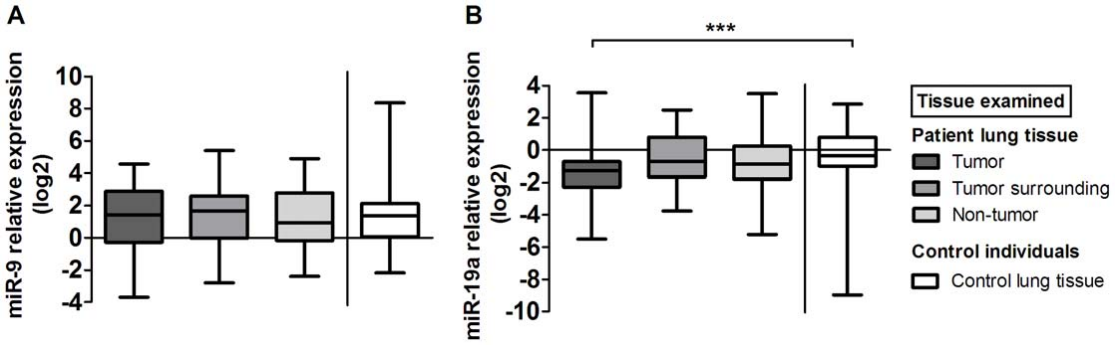
Comparative analysis of miR-9 (A) and miR-19a (B) expression in lung cancer patients' tumor tissue, surrounding tissue, and non-tumor lung tissue as well as in lung tissue from control group. Data are presented as median with range and interquartile range.

On the other side, no significant changes in miR-9 expression levels among all examined tissue samples were observed (Figure 5A).

We observed significantly lower expression of miR-19a in tumor tissue in comparison to control lung tissue (Figure 5B). The expression of miR-19a was higher in surrounding tissue form ADC patients in comparison to surrounding tissue from SCC patients (*p*<0.001; data not shown).

Since expression levels of miR-9 and miR-19a did not vary significantly between tumor tissue and corresponding non-tumor tissue, further correlations with MVD were not evaluated.

### Survival analyses

Only miRNAs with strong statistical differences in expression levels among tumor tissue versus corresponding non-tumor tissue (let-7b and miR-126) were included in survival analysis.

Median PFS for the entire group of patients was 154 days, while median OS was 236 days. No significant differences in PFS or OS for the entire group of patients were observed when patients were stratified according to histological type of lung cancer or disease stages (data not shown).

**Figure 6:**
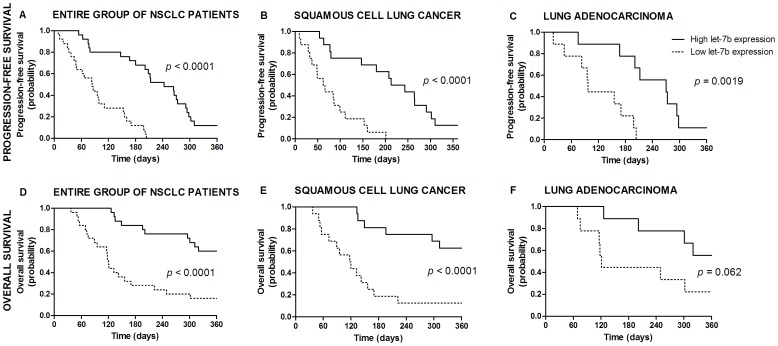
Survival curves according to let-7b expression in tumor tissue. Progression-free survival in the entire group of NSCLC patients (A), in patients with squamous cell lung cancer (B) and adenocarcinoma (C). Overall survival in the entire group of NSCLC patients (D), in patients with squamous cell lung cancer (E) and adenocarcinoma (F).

**Figure 7:**
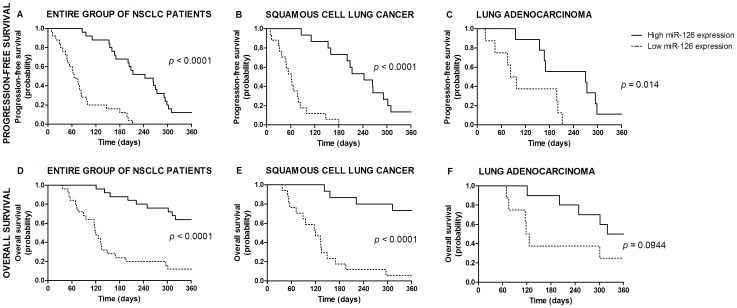
Survival curves according to miR-126 expression in tumor tissue. Progression-free survival in the entire group of NSCLC patients (A), in patients with squamous cell lung cancer (B) and adenocarcinoma (C). Overall survival in the entire group of NSCLC patients (D), in patients with squamous cell lung cancer (E) and adenocarcinoma (F).

PFS and OS according to either MVD status or miRNA expression for lung cancer patients are presented in Table 1 and Figures 6–7. Briefly, low expression of let-7b, miR-126 or both miRNAs combined was correlated with short PFS or OS in the entire group of lung cancer patients. By adopting the significance level at 0.01, the correlations remained significant in a subgroup of SCC patients, while a trend towards shorter PFS or OS was observed in a subgroup of ADC patients.

**Table 1:** Progression-free survival (PFS) and overall survival (OS) according to microvessel density and miRNA expression in tumor tissue.

	Median PFS (days)		p-value^1^	Median OS (days)		p-value^1^
			HR (95% CI)			HR (95% CI)
MVD	high^2^	low^2^		high^2^	low^2^	
All patients	75	213	<0.0001	120	NR	<0.0001
			7.23 (3.47–15.07)			6.27 (2.90–13.53)
ADC	83	270	0.0004	126	NR	0.071
			10.44 (2.86–38.13)			3.12 (0.91–10.70)
SCC	62.5	210.5	<0.0001	118.5	NR	<0.0001
			6.81 (2.75–16.88)			9.58 (3.57–25.73)

let-7b expression						
All patients	243	84	<0.0001	NR	120	<0.0001
			0.14 (0.07–0.29)			0.20 (0.09–0.43)
ADC	270	97	0.0019	NR	121	0.062
			0.14 (0.04–0.49)			0.31 (0.09–1.06)
SCC	228	64	<0.0001	NR	118.5	0.0002
			0.15 (0.06–0.37)			0.16 (0.06–0.42)

miR-126 expression						
All patients	243	66	<0.0001	NR	120	<0.0001
			0.10 (0.04–0.21)			0.14 (0.06–0.31)
ADC	270	90	0.014	342.5	122	0.094
			0.22 (0.07–0.74)			0.33 (0.09–1.21)
SCC	191	78	<0.0001	NR	120	<0.0001
			0.07 (0.03–0.18)			0.10 (0.04–0.26)

let-7b+miR-126	high/high^2^	low/low^2^		high/high^2^	low/low^2^	
All patients	NR	48.5	<0.0001	NR	92	<0.0001
			0.05 (0.02–0.14)			0.05 (0.02–0.16)
ADC	NR	83	0.017	NR	116	0.036
			0.09 (0.01–0.65)			0.14 (0.02–0.88)
SCC	NR	48	<0.0001	NR	89	<0.0001
			0.04 (0.01–0.16)			0.04 (0.01–0.16)

Footnote: MVD: microvessel density; ADC: lung adenocarcinoma; SCC: squamous cell lung cancer; PFS: progression-free survival; OS: overall survival; NR: not reached due to 1 year observation time; HR: hazard ratio; CI: confidence interval.

1 Log-rank test;

2 groups with high/low MVD or high/low expression for selected miRNAs according to cut-off values (median MVD or median relative expression).

## Discussion

Molecular mechanisms that underlie the complex regulation of tumor angiogenesis are poorly understood. However, an increasing body of evidence in recent years indicates miRNAs as important regulators of physiological and pathological angiogenesis. To evaluate angiogenic role of selected miRNAs, their expression levels were determined and correlated with MVD in NSCLC patients. We demonstrated a significantly decreased expression of two miRNAs (let-7b and miR-126) and higher degree of MVD in tumor tissue and surrounding tissue in comparison to corresponding non-tumor tissue. No differences were observed in MVD and expression levels of both miRNAs between tumor tissue and surrounding tissue from NSCLC patients. The expression of both miRNAs also negatively correlated with MVD and was significantly associated with disease prognosis. These observations strongly suggest a possible anti-angiogenic role of let-7b and miR-126 in lung cancer development. Moreover, the regulation of let-7b and miR-126 expression could have therapeutic potential because it might reduce tumor angiogenesis and therefore suppress tumor growth in lung cancer patients.

Angiogenesis could be evaluated by different methodological approaches. In our study, we used MVD as an indicator of the angiogenic potential of tissue samples. Our results indicate high MVD in tumor tissue and surrounding tissue in comparison to non-tumor tissue from NSCLC patients or the control group. We found no significant differences between tumor tissue and surrounding tissue. These observations are consistent, although they are not directly comparable with the findings of other studies, suggesting that the microenvironment of surrounding tissue exerts an important potential for tumor development, maintenance, and progression [26]–[28]. So far, several studies have reported high expression of either pro-angiogenic factors (e.g., transcription factor HIf-1α, growth factors VEGF and bFGF) or factors involved in vascular development and remodeling (e.g., endoglin CD105) in tumors and/or surrounding tissue [26], [28]–[30]. Various types of stromal cells, such as endothelial cells, smooth muscle cells, fibroblasts, and infiltrating cells of the immune system within the tumor microenvironment have been found to produce these pro-angiogenic mediators, thereby promoting crucial events in tumor growth and angiogenesis [27], [31], [32].

This study showed a significantly lower expression of let-7b and miR-126 in tumor tissue when compared to either non-tumor tissue from NSCLC patients or lung tissue from the control group. Our results are consistent with other published studies, where reduced expression of let-7 family, including let-7b [13], [15], [16] or miR-126 [9], [12], [15], [19], [20], [33] in tumor tissue or tumor cell lines of various human cancers has been reported. Expression levels of both let-7b and miR-126 were also significantly lower in surrounding tissue, where no tumor cells were present. To our knowledge, direct comparison of let-7 or miR-126 expression levels between tumor tissue and surrounding tissue has not been reported. On the basis of our results, one may conclude that similar molecular changes are present within tumor tissue and its surrounding tissue. Furthermore, a strong negative correlation between MVD, an indicator of tissue angiogenic potential and expression of let-7b or miR-126 in all examined tissue samples was observed, strongly implying their role in suppressing angiogenic processes. Moreover, these observations again indicate the great importance of the tumor microenvironment in cancer development and its involvement in tumor angiogenesis.

Furthermore, survival analysis of both miRNAs showed, that NSCLC patients with low expression of either individual miRNA or both miRNAs combined (let-7b and miR-126) were highly associated with poor survival outcomes (PFS and OS). However, the associations were significant in a subgroup of SCC patients, although ADC patients with low expression of both miRNAs in tumor tissue tended to have shorter PFS or OS. This strong impact of let-7 or miR-126 expression on disease prognosis has been reported by other research groups [13], [15], [34]–[36]. Several studies correlated reduced expression of let-7 in tumor tissue with poor survival outcomes of lung cancer patients [13], [15]. Similarly, the relationship between low miR-126 expression in tumor tissue and worse disease prognosis has been reported in glioblastoma [34], breast cancer [35] and gastric cancer patients [36]. As opposite to our results, Donnem et al. [37] demonstrated that high miR-126 expression in tumor samples correlates with a shorter survival of NSCLC patients, especially in those with squamous cell carcinoma. Even more interesting, a positive correlation between miR-126 and VEGFA expression was observed, which suggests a possible positive regulatory role of miR-126 in tumor angiogenesis [37].

Even more important, Takamizawa et al. [13] demonstrated that growth of lung cancer cells could be inhibited by introduction of expression constructs overexpressing let-7. Altogether, these findings could be potentially used for developing novel cancer-targeted therapies, which would restrict side effects, subsequently improve life quality and prolong overall survival rates.

This study found no significant differences in miR-9 expression levels among all examined tissue samples and no association with MVD. Nevertheless, larger studies are warranted to determine the exact role of miR-9 expression in tumor angiogenesis, since only a limited number of studies in lung cancer have been published so far [15], [38].

Our results indicate a lower expression of miR-19a in tumor tissue in comparison to other tissue samples. These findings are conflicting and oppose the pro-angiogenic role of miR-19a reported by the majority of studies published so far [14], [23], [25], [39], [40]. However, several authors reported that individual members of the miR-17-92 cluster, including miR-19a, might have different functions in cancer cell growth and angiogenesis [14], [39], [40]. Moreover, TSP-1, which has been described as one of the major targets of miR-19a, could either reduce [23] or, after binding with cell surface receptors integrins, even promote angiogenesis [41]. Nevertheless, further investigations of individual members of the miR-17-92 cluster and their involvement in angiogenic processes are needed.

In conclusion, our results indicate that significantly decreased expression of let-7b and miR-126 in tumor tissue has an impact on prognosis of NSCLC patients. These findings could provide us an important step toward understanding the complex pathways necessary for development and progression of lung cancer. Moreover, miRNAs have been proposed as attractive targets for developing treatment strategies that would achieve better clinical outcomes in cancer patients. However, the present results provide only for conclusions based on correlative analysis and further validation through mechanistic studies seems mandatory.

## Materials and Methods

### Patients and tissue samples

This multi-centric study was begun after it was approved by the Human Research Ethics Committee of Bosnia and Herzegovina and the corresponding organization in Serbia and all patients gave their informed written consent. Collection and subsequent preservation (formalin fixation, paraffin embedding) of tissue samples from the participating patients and controls as well as immunohistochemical evaluation were performed at General Hospital Tešanj (Bosnia and Herzegovina) and the Clinic for Pulmonary Diseases in Kruševac (Serbia). Molecular laboratory procedures including RNA extraction, reverse transcription, and real-time PCR expression analysis on tissue samples were performed at the University Clinic Golnik (Slovenia).

50 patients with non–small–cell lung cancer (NSCLC), including SCC and ADC (Table 2), and 45 age- and sex-matched individuals with no clinical evidence of a malignant disease (control group) were included (Table 3).

**Table 2:** Demographic data and clinical characteristics of NSCLC patients and control group.

Characteristic		All patients,	Adenocarcinoma,	Squamous carcinoma,
		*N* (%)	*N* (%)	*N* (%)
		50 (100)	18 (36)	32 (64)
Age in years, median (range)		63 (41–84)	62 (46–77)	64 (41–84)
Sex	Female	10 (20)	15 (83)	25 (78)
	Male	40 (80)	3 (17)	7 (22)
Active smokers		50 (100)	18 (100)	32 (100)
Clinical stage of disease	II	5 (10)	1 (6)	4 (13)
	III	26 (52)	9 (50)	17 (53)
	IV	19 (38)	8 (44)	11 (34)

**Table 3:** Demographic data and clinical characteristics of control group.

Characteristic		*N* (%)
**Control group**		45 (100)
Age in years: median (range)	60 (30–80)	
Sex	Female	15 (33)
	Male	30 (67)
Active smokers		40 (89)
	No pathological finding	21 (47)
	Acute inflammation	9 (20)
	Chronic inflammation	8 (18)
	Mild dysplasia	7 (15)

For pathological examination, immunohistochemistry, and miRNA analysis, forceps biopsies of tumor tissue, surrounding tissue (2 cm radius around primary tumor tissue without malignant cells), and patients' non-tumor lung tissue were taken from patients with lung cancer. The diagnosis of SCC or ADC in all tumor tissue samples was confirmed by an experienced pathologist before enrolment into the study. We included tumor tissue samples with at least 70% tumor tissue, as confirmed by pathohistology, and surrounding tissue as well as non-tumor lung tissue with no pathological sings of any cellular malignant changes. One forceps biopsy was taken from control individuals without any clinical evidence of a cancerous disease (control group). Tissue samples were immediately formalin fixed and than paraffin embedded using standard procedures.

### Immunohistochemistry

Sections of 5 µm were cut from paraffin-embedded tissue blocks. From each tissue specimen, sections stained with hematoxylin-eosin were used for morphological examination and quality assessment. Immunohistochemical analysis of vascularization was performed using the analySIS system and the monoclonal antibody against Factor VIII (DAKO, Glostrup, Denmark). Briefly, 5 µm thick FFPE sections were cut, placed on slides coated with 3-triethoxysilylpropylamine (Sigma, St. Louis, Missouri, USA), and fixed at 37°C overnight. After deparaffinizing in xylene and rehydrating through graded alcohols, the slides were incubated in H_2_O_2_ to block endogenous peroxidase activity. Then sections were incubated for 1 hour with primary monoclonal antibody against Factor VIII (dilution 1∶1200) at room temperature. After washing, the sections were incubated with biotinylated goat anti-rabbit immunoglobulin (dilution 1∶300; Vector Laboratories, Burlingame, California, USA), washed again, and after addition of 3,3′-diaminobenzidine tetrahydrochloride the peroxidase activity was visualized. Hematoxylin was used as a counter stain.

The degree of vascularization was measured by the average number of Factor VIII-positive microvessels in three different areas at 200-fold magnification and recorded as microvessel density (MVD). Briefly, the Factor VII stained sections were initially scanned at low power (100-fold magnification) and the areas having the highest number of microvessels (hot spots) were selected. Subsequently, microvessel counting was performed in three different areas at 200-fold magnification and the mean value was used for further analysis. Any clearly stained endothelial cells or cell clusters were considered as a single countable microvessel, regarding the presence of lumen and large vessels were automatically excluded from the analysis.

### Selection of specific miRNA involved in angiogenesis

To select miRNAs involved in cancer angiogenesis regulation, available publications and several target prediction programs/databases were searched, including miRanda (http://cbio.mskcc.org/cgi-bin/mirnaviewer/mirnaviewer.pl), TargetScan (http://www.targetscan.org/), PicTar (http://pictar.mdc-berlin.de/), and miRbase (http://microrna.sanger.ac.uk/). Based on these findings, four miRNAs (specifically, let-7b, miR-9, miR-19a, and miR-126) were selected. Selected miRNAs were shown to have roles in angiogenesis, either directly or indirectly repressing the translation of crucial angiogenic factors such as VEGFA, TSP-1, CTGF, vascular cell adhesion molecule 1 (VCAM-1), fibroblast growth factor (FGF), E-cadherin (CDH1), MMPs (matrix metalloproteinases), and tumor growth factor (TGF).

### RNA extraction, reverse transcription, and real-time PCR

Total RNA was extracted from 10 FFPE tissue sections 5 µm thick using the miRNeasy FFPE Kit (Qiagen GmbH, Hilden, Germany) following the manufacturer's instructions. Total RNA concentration was assessed using the Quanti-iT RNA Assay Kit (Invitrogen, Carlsbad, CA, USA). Two step real-time PCR was used to analyze the expression of selected miRNAs. In the first step total RNA was reverse transcribed using miRNA-specific RT primers (RNU6B, hsa-let-7b, hsa-miR-9, hsa-miR-19a, and hsa-miR-126) and a TaqMan® MicroRNA Reverse Transcription Kit (Applied Biosystems, Foster City, California, USA). The RT reaction was performed in 10 µl volume, containing 1 µl Taqman RT Buffer (10×), 0.10 µl 100 mM dNTPs, 0.67 µl MultiScribe Reverse Transcriptase (50 U/µl), 0.13 µl RNase inhibitor (20 U/µl), 2 µl specific miRNA primer, 3,33 µl of RNA, and nuclease free water to make the final volume 10 µl. Products of RT reaction were used in a real-time PCR reaction with the TaqMan miRNA assays (Applied Biosystems) and TaqMan Universal Master Mix II, no UNG (Applied Biosystems), according to manufacturer's instructions. Briefly, 1.33 µl cDNA was mixed with 5 µl TaqMan Universal Master Mix II, no UNG (2×), 0.5 µl TaqMan miRNA assay and 3.17 µl nuclease-free water. All samples were run in triplicate. The RNU6B (Applied Biosystems), was used as an endogenous control for normalization of the target miRNAs and a sample from the control group was used as a calibrator. Relative expression was calculated using the ΔΔCt method. The RQ was determined by 2^−ΔΔCt^, where ΔCt = (average of triplicate Ct_Target miRNA_−average triplicate Ct_RNU6B_) and ΔΔCt = (ΔCt_Sample_−ΔCt _Sample from control group_). Real-time PCR was performed on ABI PRISM 7500 Real-Time PCR System (Sequence Detection System instrument equipped with SDS version 1.3.0 software; Applied Biosystems).

### Statistical analyses

The distribution of data was determined using the D'Agostino and Pearson omnibus normality test. The strength of association between miRNA expression levels, MVD and other clinical variables was analyzed with the Mann-Whitney U-test, unpaired t test or Fisher's exact test as appropriate. The Spearman rank correlation test was used to analyze the degree of linear association between MVD and relative expression of individual miRNA. Patients were dichotomized into groups with high/low MVD or high/low expression for selected miRNAs in tumor tissue according to cut-off values (median MVD or median relative expression, respectively). All patients included in this study were observed for 1 year; after this time survival probabilities (PFS and OS) were calculated from the day of diagnosis to the time of event (progress or death, respectively) or loss to follow-up using the Kaplan-Meier method and the log-rank test was used to compare different categories. Statistical analyses were performed using GraphPad Prism 5 software (San Diego, California, USA) and probability values of *p*<0.01 were accepted as significant according to Bonferroni correction for 5 tests. Statistically significant differences are presented as: ** *p*<0.01; *** *p*<0.001.
